# Data on the impact of peripheral artery disease and of type 2 diabetes mellitus on the risk of cardiovascular events

**DOI:** 10.1016/j.dib.2018.10.153

**Published:** 2018-11-03

**Authors:** Christoph H. Saely, Marc Schindewolf, Daniela Zanolin, Christine F. Heinzle, Alexander Vonbank, Guenther Silbernagel, Andreas Leiherer, Heinz Drexel, Iris Baumgartner

**Affiliations:** a Department of Medicine I, Academic Teaching Hospital Feldkirch, Feldkirch, Austria; b Vorarlberg Institute for Vascular Investigation and Treatment (VIVIT), Feldkirch, Austria; c Private University of the Principality of Liechtenstein, Triesen, Liechtenstein; d Division of Angiology, Swiss Cardiovascular Center, University Hospital Berne, Berne, Switzerland; e Drexel University College of Medicine, Philadelphia, PA, USA

## Abstract

Here, we provide additional data addressing the individual and combined associations of type 2 diabetes (T2DM) and of peripheral artery disease (PAD) with future cardiovascular events in a prospective cohort study including 338 PAD patients and 711 patients who did not have PAD. Subgroup analyses regarding patient age as well as additional Cox regression models taking into account medications are presented. This data article is related to a research article titled “Single and combined effects of peripheral artery disease and of type 2 diabetes mellitus on the risk of cardiovascular events: a prospective cohort study” (Saely et al., 2018).

**Specifications table**

**Table:** 

Subject area	Medicine, clinical research
More specific subject area	Vascular medicine, epidemiology
Type of data	Figure, tables
How data were acquired	National registries, blood pressure recordings, body weight and height measurements, standard laboratory procedures for the measurement of biochemical variables, review of patient registries; data were entered into Microsoft Access and evaluated using the software package SPSS 24.0 for Windows (SPSS, Chicago, IL, USA).
Data format	Analyzed data
Experimental factors	Prospective cohort study including 1049 subjects, 338 with peripheral artery disease and 711 without peripheral artery disease. These patients were prospectively followed, and cardiovascular events were recorded.
Experimental features	Over a mean of 7.2 years cardiovascular events were prospectively recorded in 1049 subjects, encompassing 4 groups: 558 with neither PAD nor diabetes, 153 with T2DM but without PAD, 192 with PAD but without T2DM and 146 with the combination of PAD and T2DM.
Data source location	Feldkirch, Austria and Berne, Switzerland
Data accessibility	Data are included as a collated file
Related research article	Christoph H. Saely, Marc Schindewolf, Daniela Zanolin, Christine F. Heinzle, Alexander Vonbank, Guenter Silbernagel, Andreas Leiherer, Heinz Drexel, Iris Baumgartner: Single and combined effects of peripheral artery disease and of type 2 diabetes mellitus on the risk of cardiovascular events: a prospective cohort study (Atherosclerois 2018; in press) [1]

**Value of the data**

• The data presented here clarify the individual and combined associations of T2DM and of PAD with future cardiovascular events and thus help to better understand the cardiovascular risk associated with these entities.
• The data presented here show that age does not significantly affect the impact of T2DM on cardiovascular event risk in patients with or in subjects without PAD and thus help to understand the role of age for the cardiovascular risk associated with T2DM and PAD.
• The data presented here show that major cardiovascular medications do not significantly affect the associations between T2DM, PAD and cardiovascular events.
• The data presented here highlight the very high cardiovascular risk of PAD patients, in particular of those who additionally have T2DM. They should stimulate future epidemiologic research and provide a rationale to specifically include these patients in intervention trials aiming to reduce cardiovascular events.

## 1 Data

**Fig. 1:**
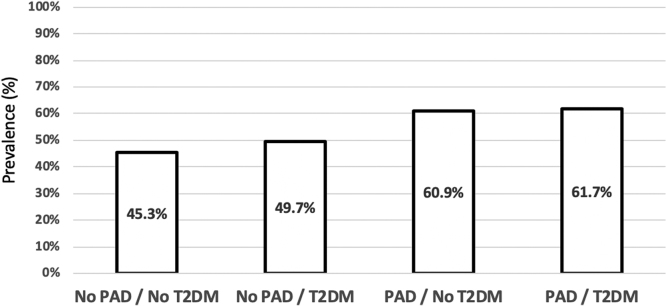
Prevalence of age ≥65 years in patient categories considering both the presence of PAD and of type 2 diabetes. From our patients, 513 were <65 years and 536 were 65 years or older. Among patients with PAD 61.2% and among subjects without PAD 46.3% were ≥65 years (*p* < 0.001). Considering both PAD and the presence of T2DM, the prevalence of an age ≥65 years was 45.3% in those with neither PAD nor T2DM, 49.7% in those with T2DM but without PAD, 60.9% in those with PAD but without diabetes and 61.7% in those with PAD plus diabetes, respectively. The prevalence of an age ≥65 years neither among subjects without (*p* = 0.341) nor among those with PAD (*p* = 0.895) was associated with the presence of T2DM.

The data presented here are related to a research article published separately by the same authors [1]. Data are shown (i) for age subgroups (Fig. 1 and Table 1) and (ii) regarding Cox regression analyses adjusting for major cardiovascular medications (Table 2).

**Table 1:** Cardiovascular risk associated with PAD and with T2DM in age-groups.

	**Age <65 years**	**Age ≥65 years**	**p**_**int**__**eraction**_
**PAD as a predictor of cardiovascular events in the total cohort**	**6.08 [3.96–9.32]**	**4.39 [3.00–6.44]**	**0.728**
**PAD as a predictor of cardiovascular events in T2DM patients**	5.40 [2.59–11.25]	10.90 [5.22–22.74]	0.115
**PAD as a predictor of cardiovascular events in patients without T2DM**	6.32 [3.60–11.09]	2.72 [1.66–4.44]	0.954
**T2DM as a predictor of cardiovascular events in the total cohort**	**1.87 [1.26–2.77]**	**1.75 [1.29–2.37]**	**0.992**
**T2DM as a predictor of cardiovascular events in PAD patients**	1.56 [0.93–2.63]	2.02 [1.37–2.98]	0.803
**T2DM as a predictor of cardiovascular events in patients without PAD**	1.45 [0.75–1.2.81]	1.09 [0.62–1.91]	0.440

Hazard ratios (HR) are shown adjusted for age, gender, BMI, smoking, hypertension, LDL cholesterol, and HDL cholesterol.

**Table 2:** Associations of PAD and of T2DM with cardiovascular events after adjustment for major cardiovascular medications.

	**Hazard ratio**
**PAD as a predictor of cardiovascular events in the total cohort**	5.07 [3.72–6.91]
**PAD as a predictor of cardiovascular events in T2DM patients**	6.73 [4.05–11.16]
**PAD as a predictor of cardiovascular events in patients without T2DM**	4.11 [2.75–6.16]
**T2DM as a predictor of cardiovascular events in the total cohort**	1.68 [1.32–2.14]
**T2DM as a predictor of cardiovascular events in PAD patients**	1.87 [1.36–2.57]
**T2DM as a predictor of cardiovascular events in patients without PAD**	1.18 [0.77–1.80]

Hazard ratios are shown adjusted for age, gender, BMI, smoking, hypertension, LDL cholesterol, HDL cholesterol and, additionally, for the use of statins, angiotensin converting enzyme/angiotensin II receptor blocking agents, beta receptor blocking agents and aspirin as well as clopidogrel.

## 2 Experimental design, materials and methods

To obtain the present data, we enrolled 1049 subjects, including 338 with PAD and 711 without PAD at the Academic Teaching Hospital Feldkirch, Austria and at the Division of Angiology at the University Hospital Berne, Switzerland from September 2006 through January 2012.

As patients with PAD, we enrolled 338 Caucasian patients who were symptomatic for PAD and had an ankle brachial index <0.9 or previous revascularization of peripheral arteries who underwent routine duplex sonography and in whom PAD was verified by sonography. Patients with type 1 diabetes or with Fontaine stage IV were not enrolled.

As subjects without PAD we enrolled individuals from a cohort of 711 consecutive Caucasian patients referred for coronary angiography for clinical reasons, in whom significant CAD with lumen narrowing ≥50% was ruled out angiographically [2] and who neither at present nor in the past had any signs or symptoms of PAD such as intermittent limb claudication, history of PAD or peripheral revascularization, or ABI <0.9). Patients with a history of acute coronary syndromes within three months prior to baseline angiography and subjects with type 1 diabetes were not enrolled.

We recorded height and weight as well as waist and hip circumferences at baseline. Data on conventional cardiovascular risk factors such as a history of smoking, hypertension, established diabetes, and a family history of atherosclerotic disease were obtained by a standardized interview. Systolic as well as diastolic resting blood pressures were measured by the Riva-Rocci method in a sitting position at the day of hospital admission after a 1 h rest. To define hypertension we used the 2013 ESC/ESH guidelines [3], and we diagnosed type 2 diabetes according to 2018 ADA clinical practice recommendations [4].

The incidence of cardiovascular events and of death was recorded during follow-up. We annually collected time and cause of death from a national registry (Statistik Austria, Vienna, Austria) as well as from hospital registries and telephone contacts; data on non-fatal events were biannually obtained using standardized interviews.

As the primary endpoint, a composite consisting of coronary death (fatal myocardial infarction, sudden cardiac death, mortality from congestive heart failure due to CAD), fatal ischemic stroke, non-fatal myocardial infarction, non-fatal ischemic stroke, and need for revascularization coronary artery bypass grafting (CABG), percutaneous coronary intervention (PCI), revascularization in the carotid or peripheral arterial beds or amputation at the lower extremities was used. Coronary angioplasty, bypass surgery, revascularizations of the peripheral arteries or amputation at the lower extremities were regarded as end points only if not scheduled as a consequence of the findings of the baseline examinations. We achieved a follow-up rate of 98.3%.

Our protocol conforms to the ethical guidelines of the 1975 Declaration of Helsinki, and the Ethics Committees of the Universities of Innsbruck and Berne approved it; written informed consent was given by all participants.

Venous blood samples were drawn after an overnight fast of at least 12 h and measurements of biochemical variables were performed from fresh serum samples, as described previously [5], [6]. Serum levels of triglycerides, total cholesterol, low density lipoprotein (LDL) cholesterol, high density lipoprotein (HDL) and cholesterol, C-reactive protein (CRP), and plasma glucose were measured on a Cobas Integra 800® (Roche, Basel, Switzerland) and Haemoglobin A1c (HbA1c) by high-performance liquid chromatography on a Menarini-Arkray KDK HA 8140® (Arkray KDK, Kyoto, Japan).

We tested differences in baseline characteristics for statistical significance with the Chi-squared and the Mann-Whitney U-tests for categorical and continuous variables, respectively. To compare differences in the cumulative incidence rates of cardiovascular events the Wilcoxon-Gehan statistic was applied. We derived adjusted hazard ratios for the incidence of first cardiovascular events from Cox proportional hazards models; continuous variables were z-transformed for these calculations. Data are given as mean (±standard deviation) if not denoted otherwise. All statistical analyses were performed using the software package SPSS 24.0 for Windows (SPSS, Chicago, IL, USA).
