# Supplementary material for: Data on the impact of peripheral artery disease and of type 2 diabetes mellitus on the risk of cardiovascular events
Source: Data Brief. 2018 Nov 3;21:1716–20. doi: 10.1016/j.dib.2018.10.153 (PMC6249514; doi:10.1016/j.dib.2018.10.153)
Supplement: Supplementary file 1 — Supplementary material [file mmc1.docx]

Berne, October 27^th^ 2018

Conflict of Interest

We wish to confirm that there are no known conflicts of interest associated with this publication and there has been no significant financial support for this work that could have influenced its outcome.

Heinz Drexel

Heinz Drexel, MD, Professor of Medicine, FESC, FAHA, FRCP

Division of Clinical and Interventional Angiology

Swiss Cardiovascular Center, Inselspital, Berne University Hospital

3010 Berne, Switzerland
